# Unveiling the Potential of Civil Briquette Furnace Slag as a Silico–Aluminon Additive in Alkali-Activated Materials

**DOI:** 10.3390/ma17246188

**Published:** 2024-12-18

**Authors:** Suoying Ren, Liang Li, Xianhui Zhao, Haoyu Wang, Renlong Zhao

**Affiliations:** 1 Department of Science and Technology, Tianjin Renai College, Tianjin 301636, China; 226511@tjrac.edu.cn; 2 School of Civil Engineering, Tianjin Renai College, Tianjin 301636, China; wanghaoyu@tjrac.edu.cn; 3 School of Civil Engineering, Hebei University of Engineering, Handan 056038, China; zhaoxianhui@hebeu.edu.cn; 4 Zhongtu Dadi International Architectural Design Co., Ltd., Shijiazhuang 050000, China; hbdd86990816@126.com

**Keywords:** alkali-activated materials, furnace slag, calcium carbide slag, mechanical strength, microstructure, aluminosilicate polymerization

## Abstract

Civil briquette furnace slag (FS), as a type of industrial solid waste, is not currently being recycled as a resource by the building materials industry. This study focuses on the potential of FS in the formulation of alkali-activated materials (AAMs) compared with calcium carbide slag (CS). This study encompasses three distinct AAM systems: alkali-activated fly ash alone (AAFA), fly ash–slag powder blends (AAFB), and slag powder alone (AABS). Electrical conductivity, fluidity, drying shrinkage, and flexural and compressive strengths were also assessed. Advanced characterization techniques, including SEM-EDS, XRD, FTIR, and TG-DSC, were utilized to examine the morphology, mineralogy, and reaction products. Despite the chemical similarity between FS and CS, FS exhibits limited active chemical components (SiO_2_, Al_2_O_3_, CaO, and MgO) and primarily functions as a physical filler, and thus lacks the chemical binding properties of CS. FS has a positive effect on the long-term compressive strength of the AABS system but not on the AAFA and AAFB systems. The NaOH-activated SP mortar sample with 20% FS reaches a compressive strength of 29.8 MPa at 360 days. The binding strength in AAMs incorporating FS is predominantly attributed to the gel formation within the alkali-activated matrix. This research offers valuable insights into the strategic use and substitution of CS, FS, and other silico–aluminon additives within the context of AAMs development.

## 1. Introduction

The industrial production of ordinary Portland cement (OPC) is a major contributor to CO_2_ emissions and depletion of natural resources [1,2]. With a current annual growth rate of 2.5% [3], the cement industry is responsible for approximately 1350 million tons of CO_2_ emissions annually [4]. In light of these environmental sustainability concerns, the construction sector is exploring alternatives to OPC, with alkali-activated geopolymer binders emerging as a promising option [5]. These binders are formed by combining silico–aluminon materials (including fly ash, slag, coal refuse, and red mud) with alkali activators (such as sodium hydroxide and sodium silicate, etc. [6]), followed by curing to produce an inorganic geopolymer binder that can match the strength of cement [7,8]. Alkali-activated materials (AAMs) generally demonstrate enhanced mechanical strength, resistance to chemical corrosion, and improved thermomechanical properties compared to conventional cement-based materials [9]. Nevertheless, the performance of these binders is contingent upon various factors, including the solid chemistry composition, concentration and dosage of alkali activators, and curing process [10,11]. Furthermore, the shift in the use of silico–aluminon materials for the synthesis of alkali-activated binders is moving away from non-renewable resources, such as metakaolin [12,13], towards recycled solid waste materials, such as fly ash and slag [14,15]. This transition not only supports the principles of deep green development in the building materials sector but also contributes to the sustainable management of industrial by-products.

Fly ash and slag serve as reactive precursors in the formation of geopolymers, a process characterized by dissolution, reorganization, and hardening under alkaline conditions, culminating in a robust three-dimensional network [16,17]. Slag, being more reactive than fly ash, generally yields alkali-activated slag geopolymers with accelerated hardening and superior compressive properties compared with those derived from fly ash [18,19]. The ratio of amorphous SiO_2_ to Al_2_O_3_ (Si/Al ratio) plays a pivotal role in gel formation and the mechanical integrity of the material [20]. The empirical formula *M_n_*[-(Si-O_2_)*_z_*-AlO_2_-]*_n_* describes alkali-activated geopolymers, where M denotes the alkali metal cations (e.g., Na^+^, K^+^, Ca^2+^) that neutralize the negative charge [21], *z* is the silicate–aluminate ratio, and *n* is the extent of polymerization, where *n* < 3 signifies a 3D network and *n* > 3 suggests a linear structure [21]. Within a Si/Al ratio of 1.0–3.0, an increased ratio correlates with an enhanced mechanical strength in alkali-activated materials [22,23,24]. Nonetheless, studies indicate that the optimal Si/Al ratio for maximum compressive strength varies with the alkali activator; for instance, a ratio of 1.6 is optimal for NaOH-activated fly ash, while 1.48 is ideal for Na_2_SiO_3_-activated fly ash [25,26]. This relationship is exemplified by the initial rise and subsequent decline in compressive strength with an increasing Si/Al ratio, underscoring the dependence of the material on this ratio. An alternative study [20] revealed the feasibility of producing geopolymers from aluminum and gray cast iron slags, with the silica–alumina weight ratio emerging as a critical factor. The ratio of 3.0 for silica to alumina produced materials with adequate workability and compressive strength suitable for construction. Thus, the Si/Al ratio dictates the extent of polymerization of AAMs, influencing their compressive strength and determining their potential applications [27]. Consequently, the Si/Al ratio significantly influences the mechanical properties of AAMs derived from solid-waste materials.

Civil briquette furnace slag, a type of silico–aluminon material, is predominantly employed as a waste material for filling roads and pits due to its extensive dispersion and low utilization rate [28,29]. Civil briquettes, which are a form of solid fuel, are produced by blending coal with additives, sulfur-fixing agents, and expansion agents, followed by crushing, mixing, and shaping. This process yields fuel with a defined geometric shape and cold–heat strength, offering efficient combustion, environmental advantages, and ease of transportation and storage. Civil briquette technology is a vital clean coal technology that mitigates coal resource scarcity and pollution concerns [30]. Regions like Beijing, Hebei, and Tianjin in China are actively promoting the use of civil briquettes. The centralized collection and comprehensive utilization of civil briquette furnace slag present a significant opportunity for green and sustainable economic and environmental benefits in the production and use of civil briquettes. The chemical composition of the civil briquette furnace slag is comparable to that of the calcium carbide slag. Calcium carbide slag is known to enhance the mechanical strength of alkali-activated fly ash materials in geopolymers [31,32] and can also act as a silico–aluminon additive to create binders for composite binders composed of soda residue, calcium carbide slag, phosphogypsum, slag, etc. [33,34,35,36]. Despite these potential applications, the influence of civil briquette furnace slag on the performance of AAMs remains an area of uncertainty.

The main objective of this paper is to investigate the potential of civil briquette furnace slag as a silico–aluminon additive in alkali-activated materials. The present investigation incorporates civil briquette furnace slag as a silico–aluminon additive into three distinct systems: alkali-activated fly ash alone (denoted as AAFA), a blend of fly ash and slag powder (termed AAFB), and slag powder alone (referred to as AABS). This study delves into the impact of furnace slag on the fresh and hardened characteristics as well as the microstructures of AAMs. This analysis aims to elucidate the effect mechanism and potential of civil briquette furnace slag compared to calcium carbide slag. Firstly, the findings of this study provide experimental evidence on the effectiveness of FS as a silico–aluminon additive, which can contribute to the broader utilization of waste materials in the construction industry. This not only promotes sustainable practices but also offers a cost-effective solution to waste management. Secondly, the study’s findings inspire further research into the potential of other waste materials as additives in AAMs, opening up new avenues for sustainable construction and waste reduction strategies.

## 2. Materials and Methods

### 2.1. Raw Materials

This study employs alkali-activated materials (AAMs), which consist of silico–aluminon precursors, alkaline activators, fine aggregates, and silico–aluminon additives. The silico–aluminon precursors, which include fly ash (FA) and slag powder (SP), serve to investigate the effects of various alkali-activation systems. The alkaline activator used is an 8 mol/L NaOH solution, which is designed to maintain a consistent variable. River sands are selected as the fine aggregates for the mortar mix. Furthermore, two commonly available silico–aluminon additives, carbide slag (CS) and civil briquette furnace slag (FS), are incorporated into the AAMs.

**Figure 1:**
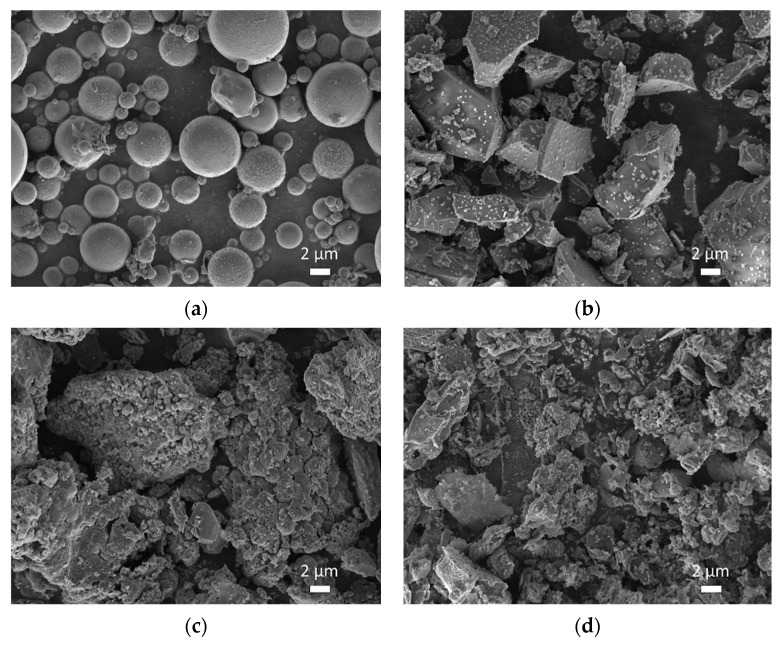
SEM images of silica–alumina precursors and additives: (**a**) fly ash, (**b**) slag powder, (**c**) calcium carbide slag, and (**d**) furnace slag.

**Figure 2:**
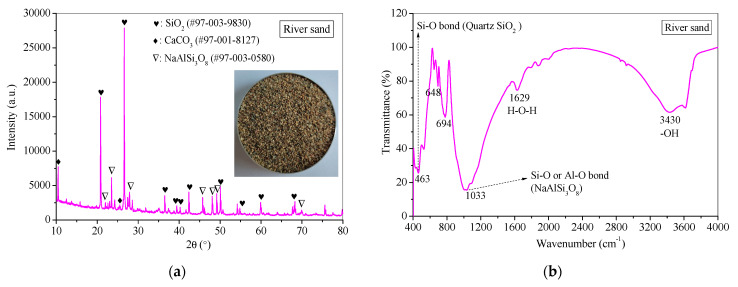
(**a**) XRD pattern and (**b**) FTIR spectrum of river sand as fine aggregate.

The chemical compositions of the binder materials, encompassing both the silico–aluminon precursors and additives, were characterized using X-ray fluorescence (XRF) analysis with a ZSX Primus III+ instrument from Rigaku, Tokyo, Japan (Table 1). The physical specifications are listed in Table 1. The morphologies of the binder materials were observed through scanning electron microscope (SEM) analysis (Figure 1). Additionally, the mineral compositions were detected via X-ray diffraction (XRD) patterns, as described in Section 3.6. To standardize the particle sizes of the two additives (FS and CS), they were processed through a ball mill, ensuring a 50% passing rate through a #325 sieve. The XRD pattern and Fourier transform infrared spectroscopy (FTIR) spectra of the river sand were also obtained for characterization (Figure 2). The utilization of raw materials in the study is as follows.

(1) Silico-aluminon precursors

The fly ash (FA) employed contains 5.32% calcium oxide (CaO), categorizing it as Class F (with CaO content less than 10%). Characterized by spherical glass beads with a smooth surface (as depicted in Figure 1a), the FA is composed of 76.52% by mass of silicon dioxide (SiO_2_) and aluminum oxide (Al_2_O_3_). The particle size distribution of FA is such that the maximum size is 10.0 μm, with an average size of 2.0 μm. Similarly, the slag powder (SP) utilized, which is a granular granulated blast furnace slag (GBFS), is composed of 51.32% SiO_2_ and Al_2_O_3_, 33.57% CaO, and 11.06% magnesium oxide (MgO). The particle morphology of SP is characterized by irregular flakes and blocks (Figure 1b). Both FA and SP are sourced from a thermal power plant located in Gongyi, Henan Province, China. These materials are recognized for their potential utility in AAM production, as evidenced by previous research [37,38].

(2) Alkaline solution

An 8 mol/L sodium hydroxide (NaOH) solution was prepared by dissolving commercially available NaOH pellets in tap water following established protocols [39,40]. These pellets, characterized by their white particulate nature, analytical purity, and purity exceeding 98%, were procured from the Kemiou Chemical Company, based in Tianjin, China. Before the preparation of the samples, the NaOH solution was allowed to cool to room temperature. The tap water employed in this study, which had a pH of 7.05 ± 0.05 and an electrical conductivity (EC) of 80.00 μS/cm, was sourced from the Economic and Technological Development Zone of Handan City, Hebei Province, China.

(3) Silico–aluminon additives

Two types of silico–aluminous additives were utilized in this study: CS and FS. Both additives possess a loose structure, which is characterized by irregularly shaped fine particles and aggregates of smaller particles that form larger units (as illustrated in Figure 1c,d). The selection of FS and CS as additives rather than precursors was based on their lower chemical reactivity compared to fly ash (FA) and slag powder (SP) [31,41]. This choice facilitates the generation of more compelling experimental outcomes within the same alkali-activated matrices. The CS is a by-product of the wet process for producing acetylene (C_2_H_2_) from calcium carbide (CaC_2_) [42]. The reaction of one ton of CaC_2_ with water results in the production of 300 kg of acetylene gas and 10 tons of waste CS, which typically contains liquid waste with a solids content of around 12%. The CS was obtained from a chemical plant in Qinghai Province, China, and is categorized as category II solid waste due to its 5.03% CaO content. In contrast, FS is a solid waste product derived from the high-temperature combustion of civil briquettes, occurring at temperatures between 800 °C and 1400 °C [29,30]. It is sourced from a chemical company in Handan, Hebei Province, China. FS is composed of 73.40% (SiO_2_ + Al_2_O_3_) and 16.10% (CaO + MgO + Fe_2_O_3_), whereas CS contains 81.00% (SiO_2_ + Al_2_O_3_) and 11.35% (CaO + MgO + Fe_2_O_3_). Despite the similarities in their main components, FS exhibits slightly higher concentrations of CaO, MgO, and Fe_2_O_3_ compared to CS.

(4) Fine aggregates

The fine aggregate used in the study was river sand, which possesses a fineness modulus of 2.62 and a bulk density of 2.64 g/cm^3^ [43]. The sand is predominantly composed of quartz (SiO₂), accounting for 92% of its chemical makeup. The presence of silicate and aluminosilicate impurities within the sand was confirmed through FTIR and XRD analysis, as depicted in Figure 2.

### 2.2. Mixing Proportion and Preparation Method of Samples

To examine the impact of FS and CS on the properties of AAMs, nine different mortar mixtures were prepared, each adhering to the mixing ratios detailed in Table 2. Prior studies highlighted the significant effect of a 20% mass ratio of CS on the physical and mechanical attributes of AAMs [31,44]. Therefore, this investigation employed a different 20% mass ratio of additives to fly ash (FA) and slag powder (SP). This ratio of 20% denoted the proportion of additives relative to the total binder materials. Control mixtures without any additives were also formulated. To evaluate the influence of FS and CS across various alkali-activation matrices, three separate systems were utilized: alkali-activated fly ash alone (AAFA), alkali-activated fly ash and slag powder blend (AAFB), and alkali-activated slag powder alone (AABS). A consistent liquid-to-solid ratio of 1:1.5 (approximately 0.67, representing the mass ratio of alkaline solution to binder materials) was maintained to ensure optimal workability of each sample at ambient temperature. It is worth noting that previous research has suggested that the highest compressive strength for AAMs based on FA is attainable within a liquid-to-binder ratio range of 0.5 to 0.7 [45,46]. Furthermore, a binder-to-sand ratio of 1:3 was maintained, which was consistent with findings from established literature [47,48].

**Figure 3:**
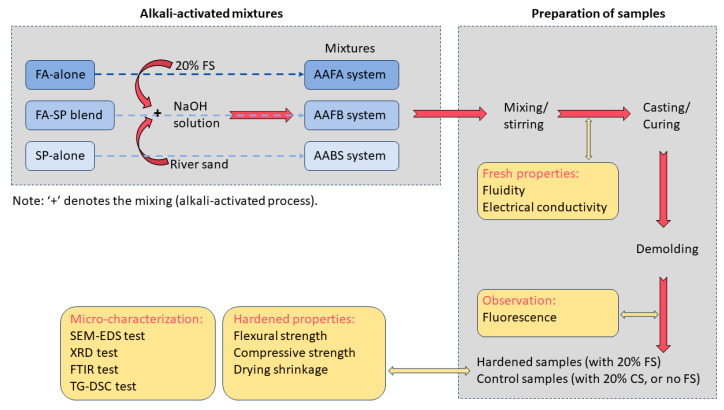
Schematic diagram of alkali-activated samples with FS and controls about the preparation process and experiment measurements.

The preparation of mortar samples involved the combination of silica–alumino precursors and additives, according to the ratios specified in Table 2, which resulted in a uniformly mixed composition (Figure 3). Subsequently, an alkaline solution was introduced and gently mixed for three minutes to create a fresh mortar paste. The fine aggregate was then added, and the mixture was stirred for an additional three minutes to achieve a consistent mortar consistency. The fresh mortar mixtures were cast into steel molds with dimensions of 40 mm × 40 mm × 160 mm and 25 mm × 25 mm × 280 mm, with the casting process involving two layers and the application of vibration to ensure even compaction. The samples were cured at room temperature (23 ± 2 °C with ≥50% relative humidity) without the use of a sealant, facilitating the observation of surface fluorescence under open-air conditions [49,50]. For sample no. FA1, which exhibited a slower rate of hardening, as indicated in Table 2, the mold was removed after three days, and curing was continued until the designated measurement age was reached.

### 2.3. Experimental Program

#### 2.3.1. Determination of Fresh and Hardened Properties

The assessment of the workability of alkali-activated mortar systems (AAMs) relies on the measurement of soluble conductive cations and ions, which are crucial for preserving the structural integrity of these materials. This evaluation is commonly achieved through the determination of electrical conductivity (EC) [31]. The viscosity and flow characteristics of AAMs are fundamental to their castability and workability, and they are integral components in the formulation of fresh AAM mixtures [51].

The quantification of EC was carried out using the DDS–307A device (LABO-HUB, Toyohashi, Japan), which is capable of measuring up to 100 mS/cm with an accuracy of 0.01 mS/cm. To maintain consistency and replicability in the methodology [51], an electrode probe with a calibration constant of 10 cm^−1^ was employed. To mitigate variations due to sample heterogeneity, EC was measured at five randomly chosen points within a single sample, and the mean value was used for subsequent analysis. The EC measurement was conducted immediately after mixing the fresh mortar and before the fluidity test, which is a critical step in the testing protocol.

After the EC assessment, the fluidity of the NLD–3 cement mortar was determined using a flow table, following the standard GB/T 2419–2005 [52]. Fresh AAM mixtures were poured into molds designed as truncated cones, and their flow diameter was measured vertically along two axes using a steel rule. The control of fluidity within the 150–300 mm range is essential to prevent premature setting in the alkali-activated slag (AABS) system, thereby guaranteeing optimal flowability.

Following the initial examination of surface fluorescence in alkali-activated samples (size of 40 mm × 40 mm × 160 mm) after demolding, and under ambient temperature and conditions, a comprehensive understanding of the hardening progression was gained over two days.

In parallel, the mechanical stability of the same samples was assessed for both short-term and long-term scenarios. This evaluation was conducted at 28 and 360 days using a servo-controlled testing machine (model DYE–300–10, manufactured by Tongli in Zuogezhuang, Hebei, China). The testing procedures adhered to the standards outlined in GB/T 17671–2021 (ISO) [48]. The compressive strength was determined at a rate of 2400 N/s, and the flexural strength was measured at 50 N/s. To ensure the consistency and reliability of the results, the mean flexural strength was calculated from at least three consecutive successful tests. The compressive strength was established by averaging the outcomes from six samples, each of which underwent multiple tests under consistent loading conditions.

Concurrently, the drying shrinkage of mortar samples (25 mm × 25 mm × 280 mm) was monitored over 360 days. After an initial curing phase of three days at room temperature, the samples and their respective molds were removed, and standardized curing conditions were implemented. The shrinkage was measured using a precision strain gauge (model BC156-300, produced by Lisheng Company, Cangzhou, China) and following the standard JC/T 603-2004 [53]. The average shrinkage values were derived from measurements taken from three parallel samples. The binder-to-sand ratio was maintained at 1:2, consistent with the standard and mirroring the proportions detailed in Table 2.

#### 2.3.2. Characterization of Microstructure and Composition

A variety of analytical tools were employed to examine the microstructural and compositional properties of AAMs. These techniques included X-ray Diffraction (XRD), Scanning Electron Microscopy with Energy-Dispersive Spectroscopy (SEM-EDS), Fourier Transform Infrared Spectroscopy (FTIR), and Thermogravimetric–Differential Scanning Calorimetry (TG-DSC).

Specifically, for the SEM-EDS analysis (Figure 3), the specimens were harvested from the core of the block samples after they had failed in compressive strength tests. This approach allowed for the examination of the samples’ internal microstructure following their failure. In a different approach, the samples intended for XRD, FTIR, and TG-DSC analyses were sourced from the same block samples but were finely milled to powder form. This method was chosen to ensure a uniform analysis and to avoid any potential directional bias that could arise from surface-specific analyses.

In the case of the SEM-EDS examination, the specimens were coated with a thin layer of gold through sputtering to improve their electrical conductivity. A detailed analysis was then performed using the TESCAN MIRA LMS SEM, which was equipped with Oxford Instruments’ Smartedx EDS system. This sophisticated setup enabled a precise assessment of the samples’ morphology and the distribution of elements within them.

The XRD analysis was conducted using a Rigaku SmartLab-SE instrument, which employed Cu-Kα radiation to delineate the crystallographic mineralogy of the specimens. The experimental parameters were set at a 40 kV tube voltage and a 40 mA current, with a scanning rate of 2° per minute. This setup enabled a thorough examination of the diffraction patterns within the 10° to 80° 2θ range, providing a comprehensive understanding of the material’s crystal structure.

For the FTIR analysis, the samples were subjected to oven-drying at 45 °C for a duration of 48 h. Subsequently, they were ground to achieve a particle size of approximately 2.0 μm. A precise sample mass of 1.0 ± 0.001 mg was then combined with 100 mg of potassium bromide (KBr) and compressed under a pressure of 20 MPa to create a pellet suitable for analysis. The analysis was performed using the Thermo Scientific Nicolet iS20 FTIR spectrometer, sourced from Wuhan, Hubei, China, which offered a resolution of 4 cm^−1^ and conducted 32 scans to interpret the spectral shifts that reflect the molecular and chemical transformations occurring within the samples.

The TG-DSC analysis was employed to investigate the thermal properties of the AAMs. This study encompassed the thermal decomposition of water and calcite, as well as the phase transitions of aluminosilicate zeolites at elevated temperatures. The STA 8000 analyzer from PerkinElmer, Waltham, MA, USA, was used for this purpose, with a sample mass of 9 to 10 mg. The samples were heated in an air atmosphere from 30 °C to 800 °C at a ramp rate of 15 °C/min, as reported in reference [54].

## 3. Results and Discussion

### 3.1. Influence of FS on Electrical Conductivity and Fluidity

**Figure 4:**
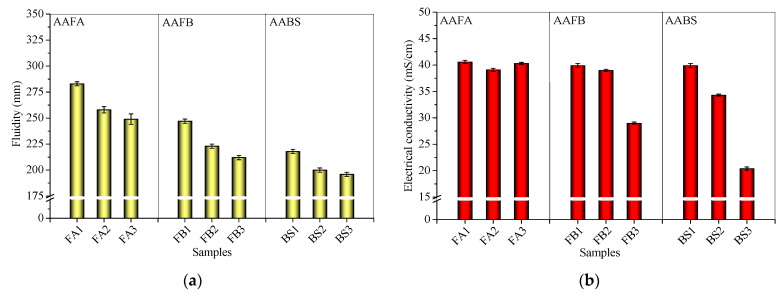
(**a**) Fluidity and (**b**) electrical conductivity of fresh alkali-activated mixtures incorporating FS or CS.

Figure 4 presents the results of fluidity and electrical conductivity (EC) of the alkali-activated systems incorporating FS or CS. The addition of 20% FS diminishes fluidity when contrasted with 20% CS, with a further decrement in fluidity as the slag powder (SP) proportion rises (Figure 4a). Comparable ECs for FA1, FB1, and BS1 are ascribed to the abundance of Na^+^ cations and OH^−^ ions in the alkaline solution. The integration of FS or CS uniformly reduces EC across AAFA, AAFB, and AABS systems, with a pronounced 48.9% decline in EC for AABS with FS, a 27.3% decrease for AAFB, and a minimal 0.7% reduction for AAFA (Figure 4b). Conversely, CS addition yields a 14.0% EC decrease for AABS, a 2.3% reduction for AAFB, and a 3.7% drop for AAFA. Notably, the same mass ratio of FS results in lower EC than CS in both AAFB and AABS.

Low water absorption of FA, when combined with NaOH, elevates mortar fluidity due to increased free water, with no substantial variation in the concentration of conductive cations and ions post-FS or CS incorporation. Conversely, high water absorption SP decreases fluidity in AAFB and AABS (Figure 4a), reducing free water and corresponding hydration reaction acceleration by soluble and conductive cations like Ca^2+^, Mg^2+^, and Na^+^ derived from FS and CS [55,56]. Both FS and CS, as by-products of high-temperature combustion, exhibit elevated absorption capacities [57], leading to an observed EC decline in AAFB and AABS systems. However, whether the addition of FS or CS can chemically promote hydration is a matter that requires further verification.

In essence, FS introduction diminishes AAMs’ fluidity and significantly lowers EC in AAFB and AABS systems, with similar effects observed from CS, highlighting the impact of high-water-absorption materials on the properties of fresh AAMs.

### 3.2. Influence of FS on Drying Shrinkage

**Figure 5:**
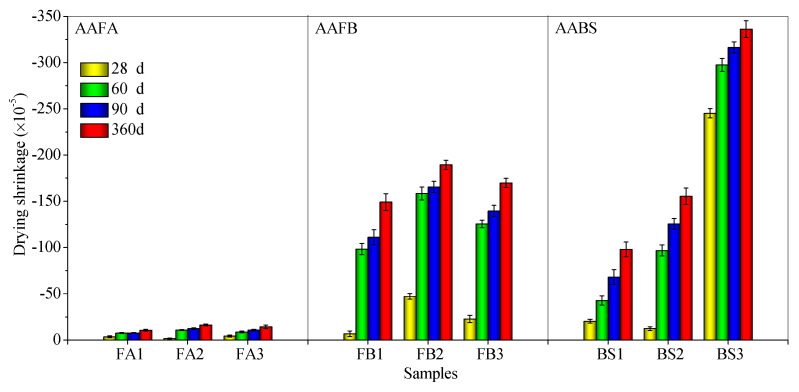
Drying shrinkage of alkali-activated mortars incorporating FS or CS with curing ages.

Figure 5 shows the drying shrinkage of alkali-activated mortars with FS or CS at 28, 60, 90, and 360 days. The drying shrinkage in AAFA systems is significantly lower than in AAFB and AABS systems at 360 days, with FA3 registering a shrinkage of 14.3 × 10^−5^. Conversely, the addition of SP to AAFB and AABS systems leads to pronounced increases, with FB3 and BS3 reaching drying shrinkages of 169.9 × 10^−5^ and 336.2 × 10^−5^, respectively. Additionally, the incorporation of 20% FS in AAFA results in a marginal reduction in 360-day drying shrinkage compared to CS (16.4 × 10^−5^). In the AAFB system, FS also reduces drying shrinkage compared to CS (189.5 × 10^−5^). However, in the AABS system, FS increases drying shrinkage beyond that of CS (155.4 × 10^−5^).

Drying shrinkage in alkali-activated systems containing FS and/or SP is attributed to the dissolution–polymerization–hardening process and hydration reactions of Al_2_O_3_, SiO_2_, and CaO components, leading to volumetric contraction as curing progresses [58,59,60]. Comparatively, FA has higher Al_2_O_3_ + SiO_2_ content, with lower levels of CaO + MgO + Fe_2_O_3_ (Section 2.1, Table 1). While CaO and MgO engage in hydration or polymerization, producing hydration products, the alkali-activated SP system requires additional Al_2_O_3_ + SiO_2_ for the formation of these products. Thus, FS with higher CaO + MgO content in the AABS system can lead to greater drying shrinkage compared to CS. In addition, Fe^3+^ in FS and CS may exist in the form of iron oxides or hydroxides, which are presumed to be embedded within the alkaline gel matrix [61,62,63]. Despite this, the dissolution of Fe^3+^ during alkali activation is minimal and evenly negligible [64,65].

In conclusion, 20% FS in AAFA and AAFB systems can achieve shrinkage rates similar to or less than those of CS. In AABS systems, the higher Al_2_O_3_ + SiO_2_ or CaO + MgO content of FS promotes additional hydration or polymerization products, increasing drying shrinkage. The chemical effects of FS are more pronounced in the AABS system.

### 3.3. Influence of FS on Flexural and Compressive Strengths

**Figure 6:**
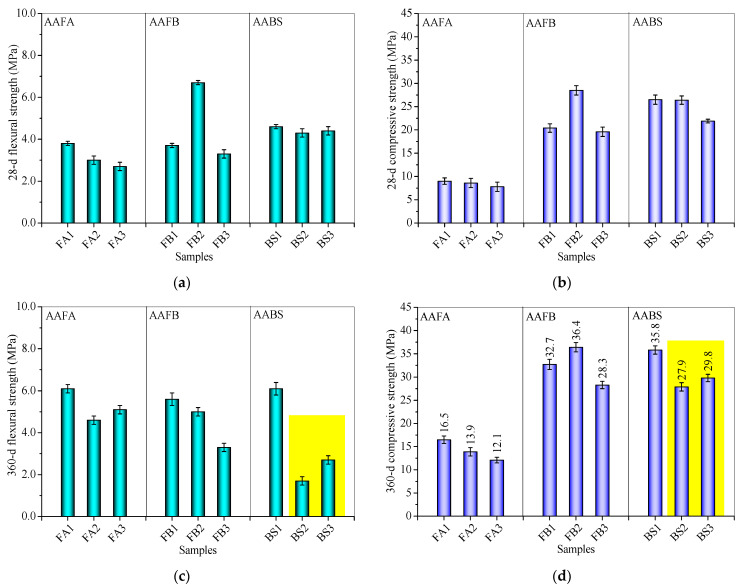
(**a**) The 28-d flexural strengths, (**b**) 28-d compressive strengths, (**c**) 360-d flexural strengths, and (**d**) 360-d compressive strengths of alkali-activated mortars incorporating FS or CS.

Figure 6 compares the flexural and compressive strengths of mortar samples at 28 and 360 days, with varying amounts of FS or CS. While the FB2 sample with CS shows increased strengths, other samples with FS or CS generally had lower early strengths compared to the control. At 28 days, 20% CS in AAFB significantly boosted flexural strength by 81.1% (Figure 6a) and compressive strength by 39.7% (Figure 6b), whereas 20% FS slightly decreased both strengths in the alkali-activated systems. Unlike CS, FS harms the early strength development of AAMs.

At 360 d, the compressive strengths increased for samples containing FS or CS, with the AAFA system showing the most pronounced gains, attributed to continued FA dissolution and gel formation enhancing bonding [66]. However, the 360 d flexural strengths of FB2, FB3, BS2, and BS3 samples were lower than at 28 d (Figure 6c) due to increased drying shrinkage (Figure 5, Section 3.2), microcrack formation, efflorescence, and other defects occurring in response to flexural stress. Long-term strength assessments reveal that in AAFA and AAFB systems, CS outperforms FS in terms of enhancing compressive strength, which is attributed to CS providing more Al_2_O_3_, facilitating the formation of aluminosilicate products, and improving particle bonding [35,41]. In contrast, FS’s contribution of additional SiO_2_ in the AABS system seems to slightly improve compressive strength (Figure 6d) due to an enhanced formation of aluminosilicate products, despite the higher initial Al_2_O_3_ and lower SiO_2_ content. This divergence suggests that the geochemical interactions of these fillers within the binder are critical, with silica availability from FS and alumina from CS influencing the polymerization process and resultant mechanical properties [67].

Furthermore, when considering the 360 d flexural and compressive strengths, the incorporation of FS into the AABS system leads to alkali-activated samples displaying mechanical strengths that are equivalent to or even better than those obtained with the addition of CS. This suggests that FS exhibits superior mechanical properties in binding alkali-activated samples. The high hydration degree in alkali-activated SP-alone systems likely plays a role, as the active chemical composition provided by CS and FS is limited, mainly serving as a physical filling function.

### 3.4. Influence of FS on Morphology and Chemical Composition by SEM-EDS Tests

**Figure 7:**
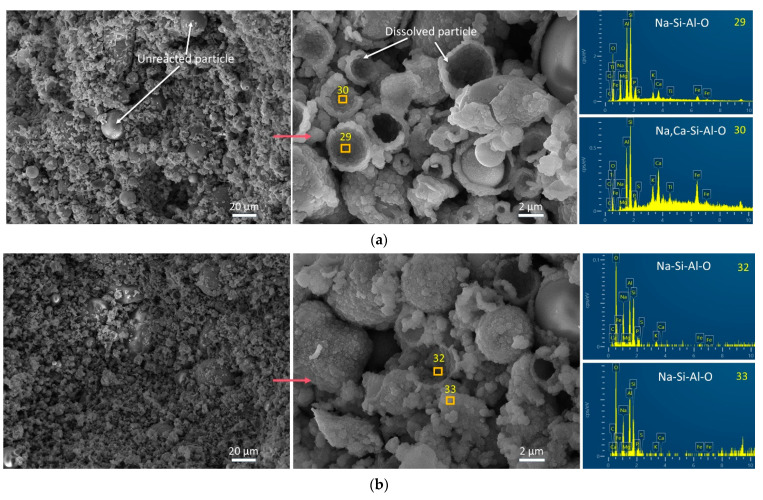

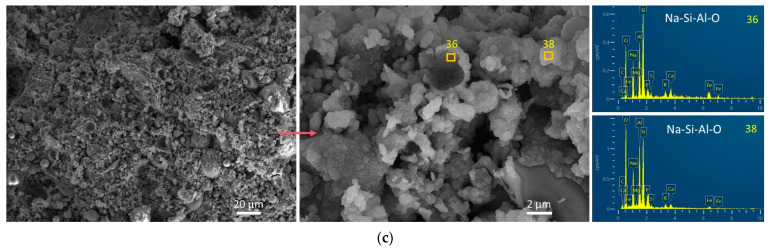
SEM images and EDS spectra of 360 d samples from the AAFA system. (**a**) FA1, (**b**) FA2, and (**c**) FA3.

**Figure 8:**
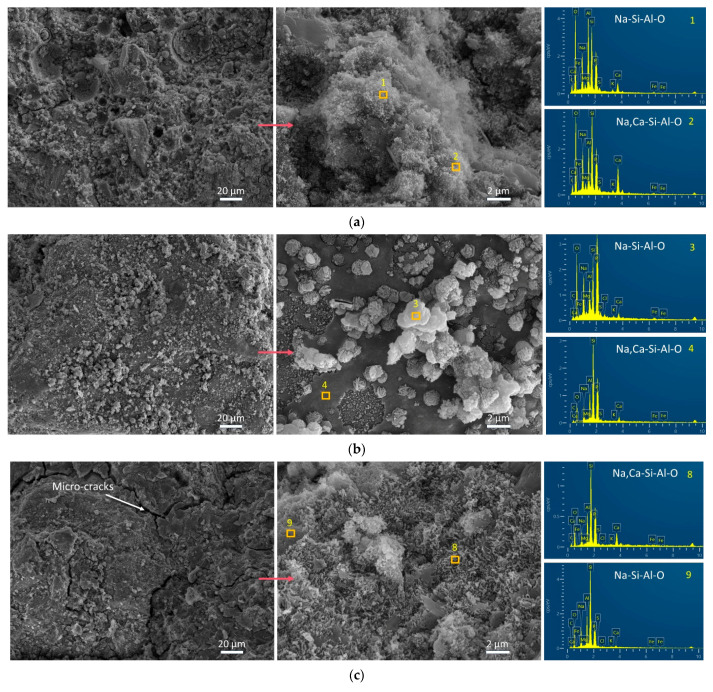
SEM images and EDS spectra of 360 d samples from the AAFB system. (**a**) FB1, (**b**) FB2, and (**c**) FB3.

**Figure 9:**
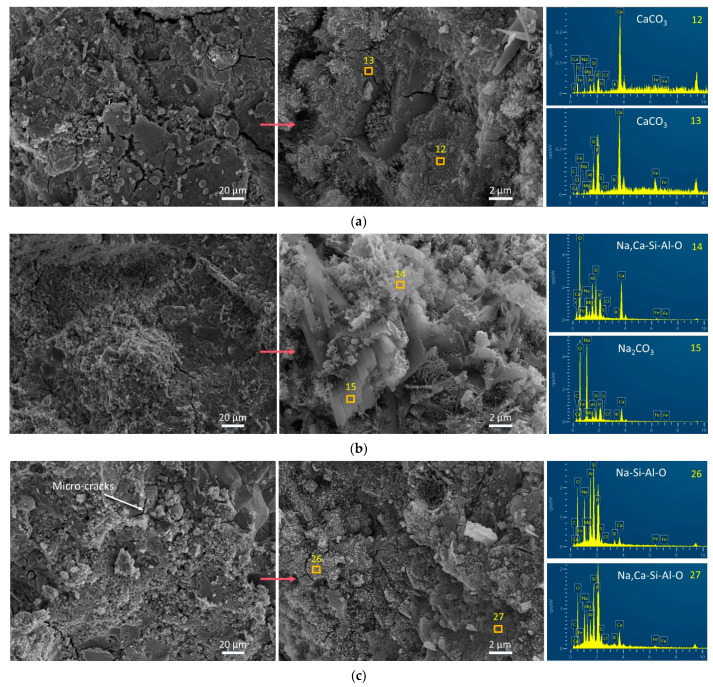
SEM images and EDS spectra of 360 d samples from the AABS system. (**a**) BS1, (**b**) BS2, and (**c**) BS3.

SEM images and EDS spectra of representative points are depicted in Figure 7, Figure 8 and Figure 9. The morphological characteristics of the AAFA system samples reveal that the addition of 20% FS or CS fails to enhance the compressive strength, as no significant structural compaction or dissolution of spherical particles is observed (Figure 7). Specifically, the AAFA samples with 20% FS or 20% CS exhibit a loose microstructure with unreacted spherical particles enveloped by gel substances, which contribute to the binder formation but compromise strength.

In contrast, FS, when combined with SP in alkali-activated systems, induces the formation of microcracks (Figure 8 and Figure 9), likely due to self-shrinkage or carbonation processes. The incorporation of SP, which possesses a high number of reactive irregular particles [68,69], results in a denser matrix and enhanced compressive strength due to accelerated hydration and gel formation [70]. Notably, the alkali-activated FA-SP system with CS (FB2 sample) demonstrates a higher degree of compactness and greater compressive strength than the corresponding FS-containing FB3 sample (Figure 8b). At room temperature, the incorporation of 20% FS fails to chemically enhance the microstructure of the AAMs. This is one of the reasons why FS is not commonly utilized as an aluminosilicate precursors but is more likely to be used as a silico–aluminon additive. Comparing the morphological characteristics of FS in Figure 7c, Figure 8c and Figure 9c with those in Figure 1d (Section 2.1), it is observed that the gel matrix of FA3, FB3, and BS3 contains unreacted, irregular FS particles. Therefore, this proves that FS has partially played a physical filling role and partially participated in the chemical hydration or polymerization process.

Furthermore, the EDS analysis of the amorphous gel products (Figure 7, Figure 8 and Figure 9) indicates a predominance of elements C, O, Na, Al, Si, K, Ca, and Fe, with trace amounts of P, S, Cl, and Mg. Heterogeneity in the AAMs is observed, as reflected by the marked difference in Fe content between different points in sample FA1 (Figure 8a). The presence of CaCO_3_ on the BS1 sample’s surface is ascribed to both the raw material SP and the reaction of excess Ca(OH)_2_ with atmospheric CO_2_ [71], as shown in Figure 9a. The introduction of FS or CS into the alkali-activated FA system leads to the formation of aluminosilicate gel products, which are predominantly composed of Na, Si, Al, and O. Similarly, when incorporated into AAFB and AABS systems, these materials also yield aluminosilicate gels with the addition of Ca.

In summary, FS was added to the alkali-activated FA and/or SP system, where it served both roles—physical filling and chemical reaction, synthesizing Na- and Ca-containing aluminosilicate polymers (Na, Ca-Si-Al-O).

### 3.5. Influence of FS on Mineral Compositions Through XRD Patterns

**Figure 10:**
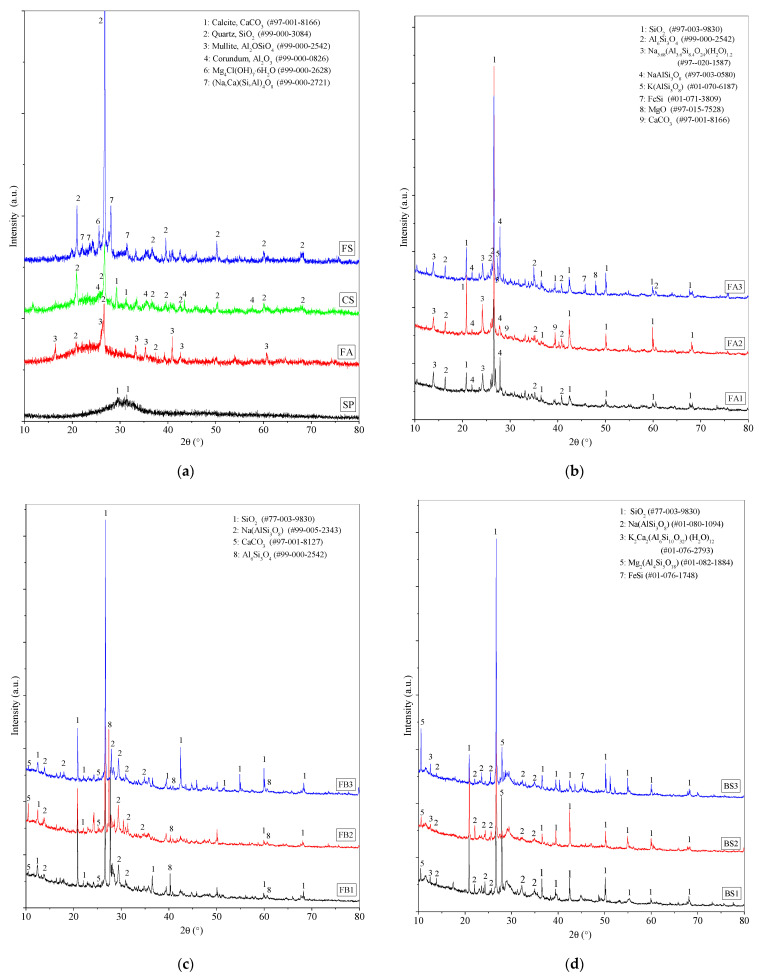
XRD patterns of (**a**) raw materials; 360 d samples from (**b**) AAFA, (**c**) AAFB, and (**d**) AABS systems.

X-ray diffraction (XRD) patterns are employed to ascertain the mineral composition of samples (Figure 10). In the raw materials, SP exhibits amorphous peaks aside from crystalline diffraction peaks of CaCO_3_ induced by carbonation [72], while the primary crystalline constituents of FA are quartz (SiO_2_) and mullite (Al_2_OSiO_4_) [73], with the remaining peaks being amorphous (Figure 10a). FS displays amorphous humps along with crystalline components such as SiO_2_, Mg_4_Cl(OH)_7_·6H_2_O, and (Na, Ca)(Si, Al)_4_O_8_. In contrast, the crystalline composition of CS predominantly consists of CaCO_3_, SiO_2_, and Al_2_O_3_. The crystalline compositions of FS and CS are generally stable, with substances undergoing chemical hydration or polymerization reactions assigned to humps between 15° and 30° 2θ. Furthermore, river sand comprises major crystalline phases of SiO_2_, CaCO_3_, and NaAlSi_3_O_8_ (Figure 2a, Section 2.1).

Upon activation of FA with a NaOH solution and curing for 360 d, the FA1 sample is obtained. The crystalline phases present in FA1 include SiO_2_, Al_6_Si_3_O_4_, Na_3.68_(Al_3.6_Si_8.4_O_24_)(H_2_O)_1.2_, and NaAlSi_3_O_8_ (Figure 10b). Specifically, SiO_2_ and NaAlSi_3_O_8_ are derived from river sand; Al_6_Si_3_O_4_ corresponds to mullite; and Na_3.68_(Al_3.6_Si_8.4_O_24_)(H_2_O)_1.2_ is a crystalline product of newly formed aluminosilicate polymers. Following the introduction of CS, the crystalline phases in FA2 exhibit an increase in CaCO_3_ as a result of the carbonation reaction between the CaO component in CS and atmospheric CO_2_. With the incorporation of FS, the crystalline phases in FA3 display an addition of K(AlSi_3_O_8_), FeSi, and MgO, compared to FA1. The combined K_2_O + Fe_2_O_3_ + MgO constituents in FS account for 10.27% and can undergo dissolution and synthesis of new products in an alkaline environment.

The activation of the FA-SP mixture with a NaOH solution yields the FB1 sample. The crystalline phases identified in FB1 include SiO_2_, NaAlSi_3_O_8_, CaCO_3_, and Al_6_Si_3_O_4_ (Figure 10c). When FS and CS are introduced, the crystalline phase compositions of FB3 and FB2 remain consistent with FB1, without the formation of new minerals. The products of aluminosilicate polymers exist in an amorphous state.

Activation of SP with a NaOH solution results in the BS1 sample. The crystalline phases present in BS1 consist of SiO_2_, NaAlSi_3_O_8_, K_2_Ca_2_(Al_6_Si_10_O_32_)(H_2_O)_12_, and Mg_2_(Al_4_Si_5_O_18_) (Figure 10d). Notably, K_2_Ca_2_(Al_6_Si_10_O_32_)(H_2_O)_12_ and Mg_2_(Al_4_Si_5_O_18_) represent newly formed aluminosilicate crystalline minerals. K_2_Ca_2_(Al_6_Si_10_O_32_)(H_2_O)_12_ is a C-A-S-H product containing K [74,75]. Mg_2_(Al_4_Si_5_O_18_) is an aluminosilicate magnesium polymer mineral formed as a result of Mg balancing the negative charge within the aluminosilicate structure. Upon the introduction of CS, the crystalline phase composition of BS2 remains identical to that of BS1; with the incorporation of FS, the crystalline phases in BS3 exhibit an additional FeSi phase compared to BS1. This indicates that, in the alkali-activated SP system, the incorporation of FS or CS allows not only Na^+^, K^+^, and Ca^2+^ ions but also Mg^2+^ cations to contribute to the formation of novel aluminosilicate products [76,77]. This corresponds to the aluminosilicate products determined by EDS spectra (Figure 9c in Section 3.4). Due to the use of mortar samples in this study, the high content of quartz (SiO_2_) complicates the detection of minor phases. Consequently, further investigation of the structure and composition of AAMs using Fourier-transform infrared spectroscopy (FTIR) is necessary.

### 3.6. Influence of FS on Chemical Bonds and Products by FTIR Spectra

**Figure 11:**
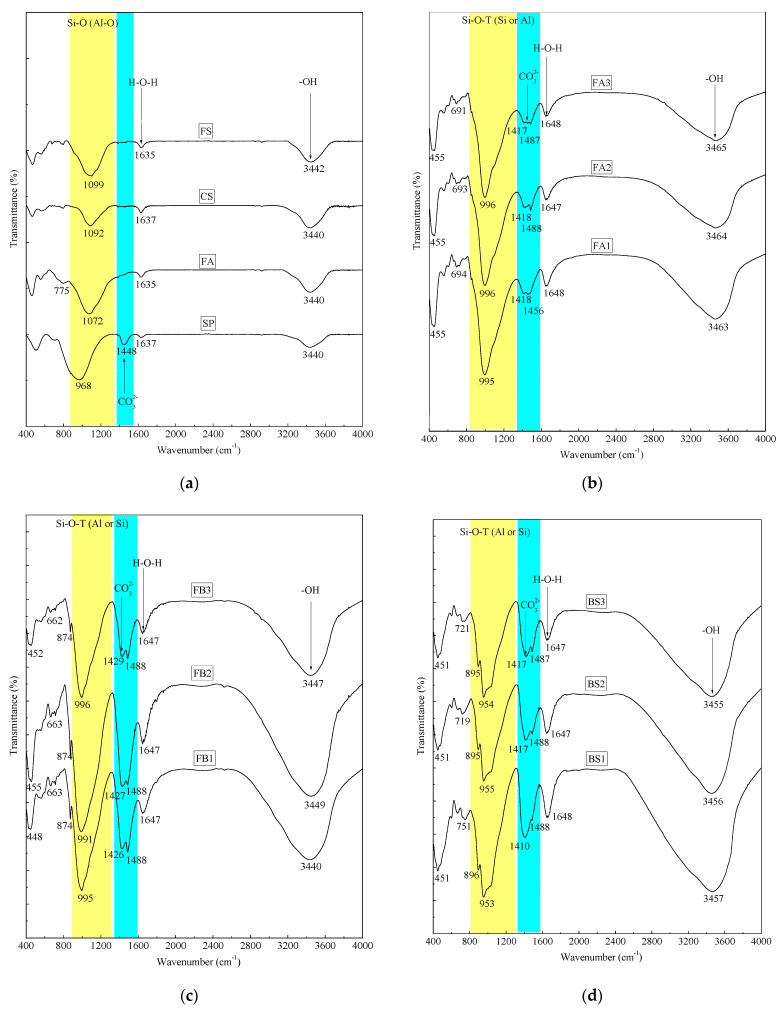
FTIR spectra of (**a**) binder materials and 360 d samples from (**b**) AAFA, (**c**) AAFB, and (**d**) AABS systems.

FTIR spectra are utilized to assess the changes in chemical bonds and products upon the introduction of FS or CS into AAMs. The incorporation of FS or CS during the alkali-activation process primarily induces shifts in the absorption peak positions associated with Si–O–T (Al or Si) and CO_3_^2−^ functional groups (Figure 11). Figure 11a illustrates the absorption peak at a wavenumber of 1448 cm^−1^, corresponding to the stretching vibrations of the CO_3_^2−^ bonds in CaCO_3_ present in SP [71,78]. Absorption peaks at 968 cm^−1^ (SP), 1072 cm^−1^ (FA), 1092 cm^−1^ (CS), 1099 cm^−1^ (FS), 984 cm^−1^ (SS), and 1033 cm^−1^ (river sand, Figure 2b) signify the asymmetric stretching vibrations of Si–O–T (Al or Si) bond [36,79].

Upon alkali activation of FA, the strong absorption peak of the Si–O–T (Al or Si) bond shifts from 1072 cm^−1^ (FA) to a lower wavenumber of 995 cm^−1^ (FA1) (Figure 11b), indicating the synthesis of Si–O–Al polymeric chains and the formation of aluminosilicate polymers [26,80]. Similarly, upon alkali activation of SP, the strong absorption peak of the Si–O–T bond shifts from 968 cm^−1^ (SP) to a lower wavenumber of 953 cm^−1^ (BS1) (Figure 11d), also suggesting the formation of aluminosilicate polymers. A comparison of the three alkali-activated systems, namely AAFA, AAFB, and AABS, reveals that a higher SP content results in a greater degree of aluminosilicate polymerization. When FS or CS is introduced into the three alkali-activated systems, the strong absorption peaks of the Si–O–T bonds show no significant changes compared to the control groups, except for a 4 cm^−1^ shift to lower wavenumbers for FB2 (Figure 11c). This indicates that the introduction of FS fails to significantly promote the polymerization of the Si–O–Al or Si-O-Si chains.

Additionally, carbonation products are detected on FTIR spectra. The strong absorption peaks at wavenumbers of 1418 cm^−1^ (FA1), 1426 cm^−1^ (FB1), and 1410 cm^−1^ (BS1) correspond to the stretching vibrations of the CO_3_^2−^ bonds in Na_2_CO_3_ or CaCO_3_ [81]. The strong absorption peaks at wavenumbers of 1456 cm^−1^ (FA1), 1488 cm^−1^ (FB1), and 1488 cm^−1^ (BS1) correspond to the stretching vibrations of the CO_3_^2−^ bonds in CaCO_3_ present in SP [78]. The strong absorption peaks of the CO_3_^2−^ bonds persist upon the introduction of FS or CS. This suggests that FS fails to mitigate the carbonation process of AAMs, but Na_2_CO_3_ as an activator favors the alkali-activated hardening process of FA-SP materials [54].

Consequently, during the synthesis of aluminosilicate gel products, FS predominantly functions as a physical filler within AAMs, despite its high content of SiO_2_ and Al_2_O_3_.

### 3.7. Influence of FS on Mass Reduction and Product Identification Through TG-DSC Curves

**Figure 12:**
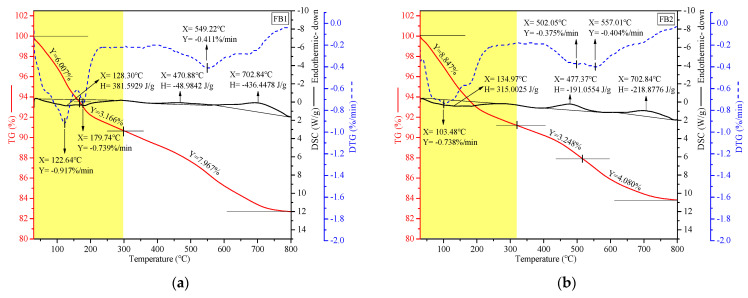

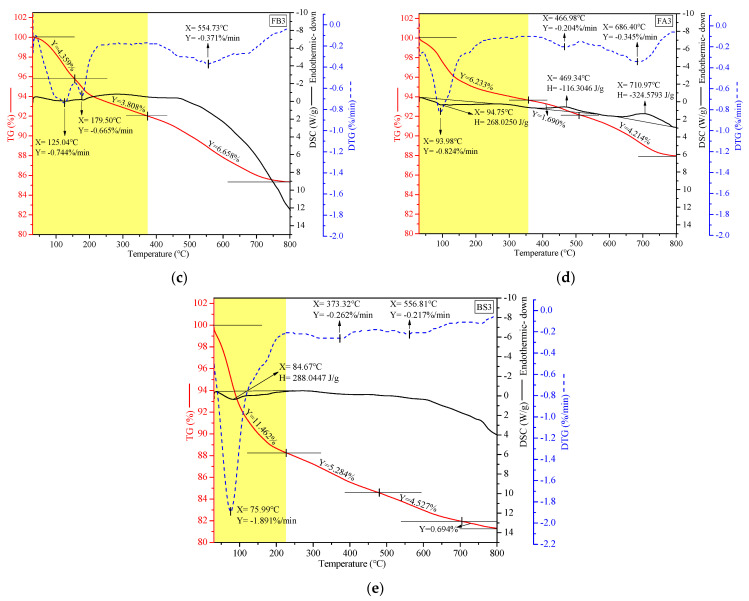
TG, DSC, and DTG curves of alkali-activated samples with FS or CS at 360 days. (**a**) FB1, (**b**) FB2, (**c**) FB3, (**d**) FA3, and (**e**) BS3.

Incorporating FS or CS into AAFA system samples results in lower compaction (Figure 7), while their introduction into the AABS system generates microcracks (Figure 9). However, when FS or CS is incorporated into the AAFB system, the flexural and compressive strengths are higher, with significant differences in strength observed (Figure 6). Consequently, further investigation of the high-temperature stability of FS or CS incorporated into AAFB system samples is warranted. To ensure comparisons were performed at the same level, each group of samples was gradually heated from 30 °C to 800 °C, yielding TG, DSC, and DTG curves. This provides information on the mass loss rate under the same level of high temperature of 800 °C [54]. The TG-DSC curves and mass loss rates of FB1, FB2, FB3, FA3, and BS3 samples are displayed in Figure 12.

The TG and DTG curves offer valuable information about the kinetic parameters and reaction mechanisms of materials [82]. This information is essential for understanding the behavior of materials under thermal conditions [83]. The DTG curve of FB1 exhibits three characteristic peaks at 122.64 °C, 179.74 °C, and 549.22 °C (Figure 12a). The peaks at 122.64 °C and 179.74 °C are attributed to the loss of interlayer water in aluminosilicate polymers containing Na and Ca [84], accounting for 9.173% of mass loss; the peak at 549.22 °C is attributed to the decomposition of CaCO_3_ into CaO and CO_2_ [44,71], accounting for 7.967%. The DTG curve of the FB2 samples, with CS incorporated, exhibits three characteristic peaks at 103.48 °C, 502.05 °C, and 557.01 °C (Figure 12b). The peak at 103.48 °C is attributed to the loss of interlayer water in aluminosilicate polymers, accounting for 8.847% of mass loss; the peaks at 502.05 °C and 557.01 °C are attributed to the decomposition of CaCO_3_, accounting for 7.328%. When FS is incorporated into the FB3 samples, the DTG curve exhibits three characteristic peaks at 125.04 °C, 179.50 °C, and 554.73 °C (Figure 12c). The loss of interlayer water in aluminosilicate polymers accounts for 8.167% of mass loss, while the decomposition of CaCO_3_ accounts for 6.658%. Therefore, at the same level of an 800 °C high temperature, the introduction of FS into the AAFB system yields a lower total mass loss compared to the introduction of CS. Additionally, the total mass loss of FB3 at an 800 °C high temperature reaches 14.825%; FA3 reaches 12.137% (Figure 12d); and BS3 reaches 17.686% (Figure 12e). The AAMs with FS incorporation and higher content of SP, however, result in slightly greater weight loss at high temperatures.

As shown by the DSC curves, FA3, FB1, and FB2 samples exhibit one endothermic peak (chemical decomposition reaction process [84]) and two exothermic peaks (physical crystallization process [44,71]); however, FB3 and BS3 samples do not exhibit exothermic peaks. This indicates that the incorporation of FS into AAFB and AABS systems is not conducive to the transformation of amorphous aluminosilicate products into crystalline forms at high temperatures.

In summary, at 800 °C, the incorporation of FS into the AAFB system significantly improves its stability, surpassing the stability achieved with CS. This finding underscores FS’s primary role in enhancing the gel’s structural stability through physical filling, aligning with the insights derived from FTIR analysis.

## 4. Discussion

In this study, 20% of either FS or CS was used as a silico–aluminon additive in alkaline-activated fly ash (FA) and/or slag (SP) materials. The effects of FS were assessed by measuring properties such as fluidity, electrical conductivity, drying shrinkage, and flexural and compressive strengths. Additionally, various chemical analysis techniques, including SEM-EDS, XRD, FTIR, and TG-DSC, were used to understand the mechanisms of FS.

The addition of FS led to a reduction in electrical conductivity and fluidity. However, it did not improve the flexural and compressive strengths of the alkali-activated materials (AAMs). Despite this, at 800 °C, FS contributed to the thermal decomposition resistance of AAMs through physical filling. Specifically, samples with FS in AAFA, AAFB, and AABS systems demonstrated compressive strengths of 12.1, 28.3, and 29.8 MPa after 360 days, respectively. Within the AAFB system, the Si/Al ratio from the multi-point analysis (Figure 8) was correlated with the compressive strength of samples after 360 days (Figure 6). The FB2 sample, which incorporated CS, exhibited a gel product Si/Al ratio of 2.97, resulting in a higher compressive strength of 36.4 MPa compared to the control FB1 sample. Conversely, the FB3 sample, modified with FS, had a gel product Si/Al ratio of 3.17, yet its compressive strength was lower at 28.3 MPa than that of FB1. The lower SiO_2_ + Al_2_O_3_ content in FS (73.4%) compared to CS (81.00%) led to a reduced amount of reactive Al_2_O_3_ being available for polymerization, thus increasing the Si/Al ratio in the aluminosilicate polymer products. When there was insufficient Al_2_O_3_ to bind with the excess reactive SiO_2_ in the system, it resulted in a diminished compressive strength when FS was introduced into the alkali-activated FA-SP materials, as opposed to the use of CS. Furthermore, the use of FS as a silicate–aluminate additive did not alter the aluminosilicate gel composition of the AAFA, AAFB, and AABS products, which are primarily composed of aluminosilicate polymers containing sodium (Na) and calcium (Ca).

The composition of the product gels formed from alkali-activated fly ash (FA) and/or slag (SP) materials plays a crucial role in determining the bonding strength of AAMs containing FS. It is important to differentiate between the composition and type of these products.

Previous studies [54,74] suggest that the ratio of FA to SP significantly influences the composition of the gel phase formed in various alkali-activated FA-SP blends. The study also notes that activator dosage and curing conditions have a lesser but still notable effect on the resulting gel chemistry [74]. It is established that in alkali-activated systems using pure SP binders, the dominant binding phase is the C–A–S–H (calcium–aluminate–silicate–hydrate) gel, in line with existing literature [85,86]. In blends containing 25% FA, the formation of a dual binding phase consisting of C–A–S–H along with the incorporation of C–(N)–A–S–H (where N can represent Na) is proposed. Increasing the FA content to 50% leads to a shift to C–(N)–A–S–H as the predominant binding phase, a trend that is supported by other studies using different alkali activators [87,88]. These findings indicate that a higher activator concentration can promote the integration of aluminum into the gel structure in the presence of FA [89].

Consequently, alkali-activated systems that include FA and/or SP, when enriched with either FS or CS, primarily yield an M-A-S-H (M–aluminate–silicate–hydrate) gel as the main product. This is primarily due to the incorporation of calcium (Ca) and magnesium (Mg) through FS and CS. In the context of the M-A-S-H gel, the ‘M’ represents alkali metal cations, which include sodium (Na), potassium (K), calcium (Ca), and magnesium (Mg). The observed phenomena are consistent with the conclusions from studies [54,74] regarding the phases C–A–S–H and C–(N)–A–S–H.

**Figure 13:**
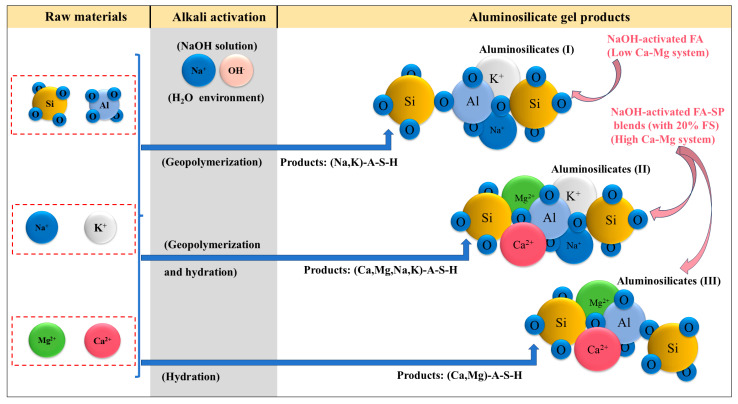
Synthesized diagram for aluminosilicate products involving the major elements of silico–aluminon precursors and additives (FS, CS, etc.).

The formation of the Si-O-Al chain in aluminosilicate structures generates structural negative charges. These charges can be balanced by alkali metal cations (M), which include Na^+^, K^+^, Ca^2+^, and Mg^2+^ [79,90]. The mineral compositions identified by XRD analysis (Figure 10), such as K_2_Ca_2_(Al_6_Si_10_O_32_)(H_2_O)_12_, Mg_2_(Al_4_Si_5_O_18_), Na_3.68_(Al_3.6_Si_8.4_O_24_)(H_2_O)_1.2_, and K(AlSi_3_O_8_), along with the alkali metal cations and main elements (Na, Ca, Si, Al, O) detected by EDS (Energy-Dispersive Spectroscopy) spectra (Figure 7, Figure 8 and Figure 9), confirm that the charge balance is achieved by alkali metal cations and that aluminosilicate products (M-A-S-H) are formed. However, the presence of excessive Ca^2+^ and Mg^2+^ cations as structural modifying cations can lead to the synthesis of (Ca, Mg)-A-S-H phases [90,91]. These modifications can affect the properties of the aluminosilicate products. The low chloride (Cl), sulfur (S), iron (Fe), and chromium (Cr) content are not considered as factors that determine the bonding strength of the materials. The mechanism by which alkali metal cations affect the aluminosilicate product gels in FS-containing AAMs is illustrated in Figure 13.

**Figure 14:**
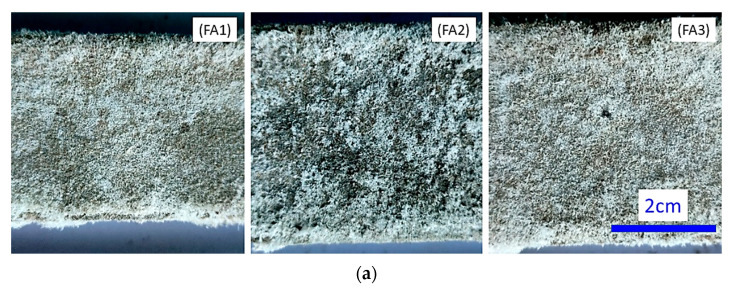

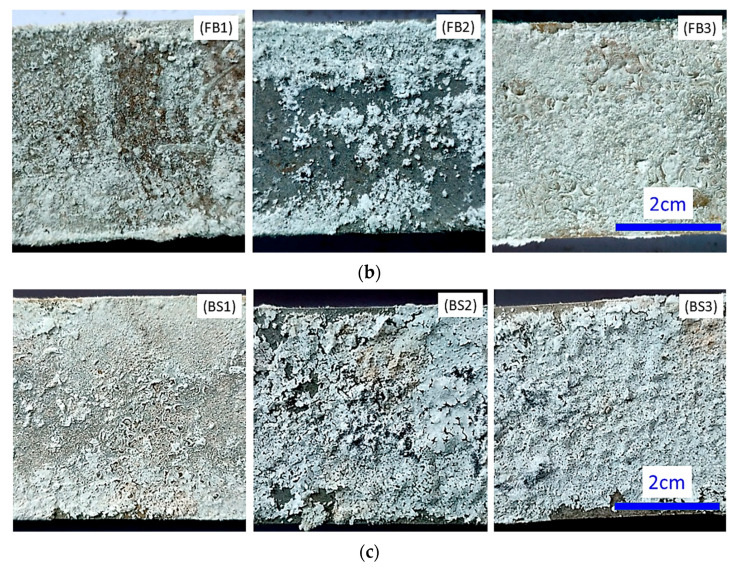
Surface fluorescence of alkali-activated samples at room temperature. (**a**) AAFA, (**b**) AAFB, and (**c**) AABS systems.

Incorporating FA or SP into AAMs can lead to surface efflorescence at room temperature (as shown in Figure 14), characterized by the formation of sodium carbonate (Na_2_CO_3_) [1]. This efflorescence was observed in SEM-EDS images of BS2 (Figure 8b) and confirmed by the absorption peak at 1410–1418 cm^−1^ in the Fourier transform infrared (FTIR) spectrum (Figure 11). The reduction in CO_3_^2−^ ion concentration results in an increase in Ca^2+^ ions, promoting the precipitation of the gel phase and reducing material porosity. Simultaneously, the reaction of Mg^2+^ ions with dissolved aluminum species leads to the formation of Mg-A-S-H, strengthening the gel matrix [54]. The presence of CaCO_3_ and Na_2_CO_3_ is beneficial for the long-term strength development of the alkali-activated FA-SP system [92]. However, a quantitative method for characterizing and evaluating efflorescence on the surfaces of alkali-activated multi-waste materials is currently lacking.

## 5. Conclusions

This work investigated the potential application of civil briquette furnace slag (FS) in alkali-activated materials (AAMs). Three alkali-activated systems were prepared: alkali-activated fly ash alone (AAFA), alkali-activated fly ash–slag powder (AAFB), and alkali-activated slag powder alone (AABS). Furthermore, a comparative analysis was conducted with calcium carbide slag (CS) of a similar chemical composition. The main conclusions obtained are as follows:

(1) FS, with its high adsorption capacity, decreased AAMs’ fluidity and electrical conductivity, particularly in AABS, with reductions of 48.9% in EC and 10.1% in fluidity.
(2) Despite causing increased drying shrinkage in AABS, the compressive strength of FS samples after 360 days was similar to or greater than that of CS samples, indicating FS’s beneficial impact on AABS’ long-term strength.
(3) FS, despite sharing a chemical composition with CS, lacks the active components for chemical bonding and primarily acts as a physical filler. Its binding strength is determined by the alkali-activated matrix’s gel products, which vary with the Ca-Mg content.
(4) Analysis shows that in the low Ca-Mg system, the gel products are Na- and K-containing aluminosilicate polymers (Na, K)-A-S-H; in the high Ca-Mg system, the gel products are Ca-, Mg-, Na-, and K-containing aluminosilicate polymers (Ca, Mg, Na, K)-A-S-H or (Ca, Mg)-A-S-H.

In summary, FS primarily serves as a physical filler in AAMs, a role which is distinct from that of CS. Consequently, as a silicon–alumina additive, FS does not effectively utilize the silicon–alumina composition and must be blended with other highly reactive silicon–alumina additives. This finding provides both theoretical and experimental guidance for the application of FS in AAMs. In addition, the study’s findings inspire further research into the potential of other waste materials as additives in AAMs, opening up new avenues for sustainable construction and waste reduction strategies.

However, the use of FS as a physical filler leads to defects such as cracking or alkali efflorescence and carbonation in the alkali-activated samples, which affect the long-term performance of the material. In the future, to further optimize the long-term durability of AAMs containing FS, it is necessary to propose methods for alkali efflorescence control and quantitative evaluation techniques. Secondly, the potential for FS to be used in combination with other waste materials or in different formulations of AAMs should be explored, as this can lead to the creation of even more sustainable and cost-effective construction solutions. Thirdly, to assess the benefits of FS recycling, a thorough examination of economic, environmental, and associated factors is crucial.

## Figures and Tables

**Table 1:** Chemical compositions and physical indexes of binder materials.

Index	Parameters	Silico–Aluminon Precursors		Silico–Aluminon Additives	
		Slag Powder (SP)	Fly Ash (FA)	Calcium Carbide Slag (CS)	Civil Briquette Furnace Slag (FS)
Chemical (mass %)	*w* (SiO_2_)	35.10	51.20	44.80	47.80
	*w* (Al_2_O_3_)	16.22	25.32	36.20	25.60
	*w* (CaO)	33.57	5.32	5.03	7.95
	*w* (MgO)	11.06	1.60	1.03	1.47
	*w* (Fe_2_O_3_)	—	2.60	5.29	6.68
	*w* (K_2_O)	—	—	0.96	2.12
	*w* (SO_3_)	—	—	3.60	5.19
	*w* (Na_2_O)	—	—	0.25	1.13
	*w* (TiO_2_)	—	—	1.76	1.12
	*w* (Ca)	23.98	3.80	3.69	5.57
	*w* (Others)	4.05	7.06	1.08	0.94
	*w* (SiO_2_ + Al_2_O_3_)	51.32	76.52	81.00	73.40
	*w* (CaO + MgO + Fe_2_O_3_)	44.63	16.52	11.35	16.10
	Si/Al (-)	1.91	1.41	1.06	1.58
	Ca/Si (-)	1.46	0.08	0.12	0.18
	pH value (-)	11.45	10.24	8.55	10.07
	EC (μS/cm)	440	1075	2700	1632
Physical	specific gravity (-)	2.67	2.45	1.80	1.10
	Amount passing #325 sieve (-)	90%	80%	50%	50%
	Specific surface area (m^2^/kg)	660	509	420	345

Note: The pH and electrical conductivity (EC) were measured at an identical solid-to-water mass ratio of 1:5 at room temperature. The data of a specific surface area are derived from the BET test, where *w* represents the mass percentage (mass%). Herein, *w* (Others) includes elements P, Cl, Cr, etc.

**Table 2:** Mixing ratios of alkali-activated fly ash (FA) and/or slag powder (SP)-based samples incorporating two additives—carbide slag (CS) or furnace slag (FS).

Systems	Sample No.	Precursors (g)		20% Additives (g)		Activator 8 M Solution (g)	Fine Aggregate (g)	Elemental Atomic Ratios (-) by XRF Method		
		FA	SP	CS	FS	NaOH	Sand	Si/Al	(Na + K)/Al	(Ca + Mg)/Si
AAFA	FA1	450	—	—	—	300	1350	1.719	0.000	0.111
	FA2	360	—	90	—	300	1350	1.543	0.011	0.113
	FA3	360	—	—	90	300	1350	1.692	0.032	0.124
AAFB	FB1	225	225	—	—	300	1350	1.766	0.000	0.483
	FB2	180	180	90	—	300	1350	1.549	0.012	0.407
	FB3	180	180	—	90	300	1350	1.723	0.038	0.417
AABS	BS1	—	450	—	—	300	1350	1.839	0.000	1.025
	BS2	—	360	90	—	300	1350	1.558	0.015	0.803
	BS3	—	360	—	90	300	1350	1.767	0.045	0.812

Note: Three distinct systems were prepared in this work: alkali-activated fly ash alone (AAFA), alkali-activated fly ash–slag powder blend (AAFB), and alkali-activated slag powder alone (AABS).

## Data Availability

The original contributions presented in this study are included in the article/supplementary material. Further inquiries can be directed to the corresponding author(s).
